# Editorial: Posterior Reversible Encephalopathy Syndrome and Associated Diseases

**DOI:** 10.3389/fneur.2020.00667

**Published:** 2020-07-24

**Authors:** Stephane Legriel, Alexander Lerner, Max Wintermark, Jeffrey Bruce Rykken, Bo Gao

**Affiliations:** ^1^Medico-Surgical Intensive Care Department, Centre Hospitalier de Versailles, Le Chesnay, France; ^2^University Paris-Saclay, UVSQ, INSERM, CESP, Team “PsyDev”, Villejuif, France; ^3^Division of Neuroradiology, Department of Radiology, Keck School of Medicine, University of Southern California, Los Angeles, CA, United States; ^4^Neuroradiology Section, Department of Radiology, Stanford University School of Medicine, Stanford, CA, United States; ^5^Department of Radiology, University of Minnesota, School of Medicine, Minneapolis, MN, United States; ^6^Department of Radiology, Affiliated Hospital of Guizhou Medical University, Guiyang, China

**Keywords:** posterior reversal encephalopathy syndrome, prognosis, pathophysiology, magnetic resonance imaging, comorbidities

Posterior reversible encephalopathy syndrome (PRES) is a clinicoradiological entity that was highlighted by Hinchey et al. (1). The typical clinical features of PRES are well-known. It associates consciousness impairment varying in severity, seizure activity, headaches, visual abnormalities, nausea/vomiting, and focal neurological signs (2). Acute hypertension is not a pathognomonic sign, although reported associated with PRES in nearly 85% of cases (3). Cerebral MRI brain is the gold standard exam to diagnose PRES. Radiological features consist in bilateral regions of edema typically but exclusively located in the white matter and predominating in the posterior parietal and occipital lobes. Indeed, frontal lobes, temporal or posterior fossa, and cortical gray matter may also be involved (4). Rarely, PRES may imply basal ganglia or even brainstem or medullary white matter (5).

PRES can develop in association with a vast array of conditions such as exposure to toxic agents (i.e., cancer chemotherapy agents, cytotoxic agents, immunotherapy, immunosuppressive agents), acute hypertension with or without underlying acute renal insufficiency, infections, preeclampsia/eclampsia, autoimmune diseases, and other miscellaneous other conditions.

The pathophysiology of PRES is not fully understood (6). There are two main hypotheses that contradict each other. The vasogenic theory involves impaired cerebral autoregulation responsible for an increase in cerebral blood flow, whereas the cytotoxic theory involves endothelial dysfunction with cerebral hypoperfusion (7). However, over the past years, several neuropathological and histological reports have been published allowing to develop further hypotheses (8, 9). Indeed, the cytotoxic theory may itself split into 3 additional hypotheses (6). The first one is the direct cytotoxic hypothesis consisting in a direct exogenic aggression when the patients are exposed to toxics drugs. The second one is the neuropeptide hypothesis, relying on an initial inflammatory aggression associating lymphocytes T and cytokines activation in cases of autoimmune diseases or in sepsis. The third one is the immune hypothesis implying endothelin 1, thromboxane A2, and prostacyclin release in case of acute hypertension, renal insufficiency, or even immunosuppressor use (10). Activation of vasopressin 1a receptors may be the common denominator of theses hypothetic mechanisms (11).

Outcomes PRES is considered to reversible once the cause is removed. However, severe complications have been reported such as brain ischemia, hemorrhages, or cerebral herniation. Thus, even if limited data are available on functional outcomes, permanent neurological impairment have been described in half of survivors after PRES requiring intensive care unit management (12). Death has been reported in up to 15% of patients (13). Finally, recurrences have been encountered in 7% of cases (14).

PRES must be diagnosed early and investigations must be performed to identify the causative factors (15). Symptomatic treatment should be given immediately, and the causative factors corrected without delay. ICU admission and life-supporting treatments may be required.

This Research Topic proposes a broad overview of PRES, bringing bench to bedside information on pathophysiology, epidemiological data, and review articles to better understand the ins and outs of this entity.

Wang et al. explored pathophysiology of PRES by reporting on an animal model of blood barrier disruption related to acute hypertension. Brain examination of sacrificed rats, after hypertension induction and demonstration of MRI features of PRES, demonstrated a significantly higher content of Evans blue than controls, indicating blood brain barrier disruption.

Hinduja et al. performed a huge overview of PRES, providing epidemiological information on both clinical and neurological features, diagnosis, but also neurodiagnostic tests that could comfort the diagnosis or explore its potential causes, prognosis, and finally management. Interestingly, the author reminds us the neuro-imaging definitions of mild and moderate PRES but also the particularly severe clinical presentation of malignant PRES. Importantly, the author concludes on the importance of large prospective studies to allow a better understanding of PRES.

Anderson et al. combine a general review of clinical and radiological findings of PRES, as well as they develop various pathophysiologic hypothesis.

Gandini et al. reported an interesting case report with literature review about a patient who experienced PRES delayed 2 months after receiving FLOT chemotherapy (5-fluorouracil, oxaliplatin, docetaxel, and folinic acid).

Zheng et al. analyzed 31 cases of cerebrospinal fluid hypovolemia responsible for PRES. The authors report epidural or lumbar puncture as the most common cause, but underline the possible implication of anesthetics and neurosurgical procedures.

Li et al. performed a reappraisal of clinical and MRI features of PRES in patients with atypical regions involvement such as basal ganglia, thalamus, periventricular or deep white matter, cerebellum, brainstem, midbrain, pons, medulla oblongata, and spinal cord. Interestingly, their systematic review concluded that common symptoms of PRES with atypical regions associated headaches (50.7%), altered mental status (43.7%), seizures (41.9%), visual disturbances (34.9%), nausea or vomiting (23.4%), and focal neurological deficits (18.2%). The underlying causes included hypertension, renal diseases, immunosuppressant drugs, and chemotherapy/chemoradiotherapy. Most cases were reversible within 2–3 weeks when properly treated.

Saad et al. performed a complementary review of imaging of atypical and complicated presentations of PRES. Thus, the authors discuss about atypical regional involvement in PRES and report on the various potential complications, namely: hemorrhage, transient or permanent cerebral ischemia, or vasospasm.

Pilato et al. proposed a didactic article on PRES and Reversible Cerebral Vasoconstriction Syndrome (RCVS) reviewing physiopathology, clinical, and neuroimaging features allowing diagnosis and prognosis of both these entities. The authors also emphasize the potential link between RCVS and PRES, an association reported in 10% of cases.

Largeau et al. demonstrated an interest in PRES of poisoning causes. By performing a systematic review, the authors identified 42 reported cases of various causes among alcohol acute/chronic intoxication or alcohol withdrawal, drug overdose, illicit drugs, natural toxin (snake bites, scorpion stings), and chemical substance abuse (organophosphorus).

Sheikh-Bahaei et al. focused their research in sweeping the spectrum of imaging techniques to better clarify the diagnosis and differential diagnosis of PRES, its complications, and thus its potential prognostic implications.

Song et al. provided further progress in PRES prognostication by evaluating the interest of combining diffusion-weighted imaging (DWI)-Alberta Stroke Program Early CT Score (ASPECTS) with fluid-attenuated inversion recovery (FLAIR) vascular hyperintensity (FVH)-DWI mismatch to discriminate the prognosis of cerebral infarction. The authors found that a DWI-ASPECTS score ≥ 8 was associated with the highest prognostic value of FVH-DWI mismatch measurement. The identification of such prognostic markers in PRES could allow to propose the evaluation of targeted therapeutic strategies.

We thank our colleagues who provided a huge effort to contribute to this very interesting Research Topic. We are also grateful to the reviewers who did not count their time allowing the production of these quality articles. We hope that this Research Topic might be a real step forward in the understanding of this intriguing and exciting syndrome.

## Author Contributions

SL wrote the original draft, assembled and incorporated comments from the co-authors, and crafted the final draft. All of the other co-authors contributed to manuscript review and revision.

## Conflict of Interest

The authors declare that the research was conducted in the absence of any commercial or financial relationships that could be construed as a potential conflict of interest.
